# Exploratory Study of the Clinical Value of Near-Infrared Sentinel Lymph Node Mapping With Indocyanine Green in Vulvar Cancer Patients

**DOI:** 10.3389/fonc.2021.652458

**Published:** 2021-04-22

**Authors:** Franziska Siegenthaler, Sara Imboden, Laura Knabben, Stefan Mohr, Andrea Papadia, Michael D. Mueller

**Affiliations:** ^1^ Department of Obstetrics and Gynecology, Bern University Hospital and University of Bern, Bern, Switzerland; ^2^ Department of Obstetrics and Gynecology, Ente Ospedaliere Cantonale, University of the Italian Switzerland, Lugano, Switzerland

**Keywords:** vulvar cancer, sentinel lymph node, indocyanine green, near-infrared imaging, Technetium-99m

## Abstract

**Background:**

This study aimed to evaluate the clinical value of indocyanine green sentinel lymph node (SLN) mapping in patients with vulvar cancer. The conventional procedure of SLN mapping in vulvar cancer includes peritumoral injection of technetium-99m nanocolloid before surgery and intraoperative injection of a blue dye. However, these techniques harbor some limitations. Near-infrared fluorescence imaging with indocyanine green has gained popularity in SLN mapping in different types of cancer.

**Methods:**

We analyzed retrospectively vulvar cancer patients at our institution between 2013 and 2020 undergoing indocyanine green SLN mapping by applying video telescope operating microscope system technology.

**Results:**

64 groins of 34 patients were analyzed. In 53 groins we used technetium-99m nanocolloid, in four patent blue, and in five both techniques, additionally to indocyanine green for SLN detection. In total, 120 SLNs were identified and removed. The SLN detection rate of indocyanine green was comparable to technetium-99m nanocolloid (p=.143) and higher than patent blue (p=.003). The best results were achieved using a combination of ICG and technetium-99m nanocolloid (detection rate of 96.9%). SLN detection rates of indocyanine green were significantly higher in patients with positive lymph nodes (p=.035) and lymphatic space invasion (p=.004) compared to technetium-99m nanocolloid.

**Conclusion:**

Indocyanine green SLN mapping in vulvar cancer is feasible and safe, with reasonable detection rates. Due to its easy application and few side effects, it offers a sound alternative to the conventional SLN mapping techniques in vulvar cancer. In patients with lymph node metastasis, indocyanine green even outperformed technetium-99m nanocolloid in terms of detection rate.

## Introduction

Inguinal lymph node status represents the most significant prognostic factor for survival in vulvar cancer patients (1). Lymphadenectomy therefore plays a crucial role in both surgical treatment and staging of vulvar cancer. Complete inguinofemoral lymph node dissection leads to a short- and long-term morbidity, consisting of wound infections or dehiscence and lymph edema in up to 50% of all patients (2). However, only one third of patients with stage I or II disease have lymph node metastasis and consequently up to two thirds undergo unnecessary lymphadenectomy (3), associated with high morbidity and prolonged hospitalization (4).

The introduction of sentinel lymph node (SLN) biopsy in vulvar cancer, first described by Levenback in 1994 (5), provides a less invasive technique for staging of vulvar cancer than complete inguinofemoral lymph node dissection, with significant reduction in lymphedema, wound infection, and dehiscence without compromising groin recurrence rates or survival rates (6, 7). SLN biopsy has been shown to be oncologically safe in unifocal squamous-cell vulvar cancer up to a tumor size of 4 cm with clinically negative lymph nodes (6, 8) and with a low false negative rate of approximately 3% (9).

The conventional technique of SLN mapping in vulvar cancer involves a peritumoral injection of technetium-99m (^99m^Tc) nanocolloid before surgery combined with an intraoperative injection of a blue dye. Preoperative 3D single photon emission tomography imaging helps detecting the SLN more precisely regarding number and anatomical localization (10). However, these techniques harbor some limitations: (a) the preoperative injection of radiotracers involves a painful procedure for the patient; (b) the transport and storage of radioactivity requires complex logistics; (c) blue dyes may lead to staining of the injection site and to allergic reactions; and (d) visualization of the blue dye is limited when the lymphatic tissue is covered by skin or fat – resulting in a lower detection rate.

In cervical and endometrial cancer, SLN mapping with near-infrared fluorescence imaging using indocyanine green (ICG) has shown better overall and bilateral detection rates as compared to a combination of blue dye and ^99m^Tc-nanocolloid, a better safety profile than blue dyes, and an easier application than ^99m^Tc-nanocolloid (11–14). Furthermore, ICG and near-infrared fluorescence imaging outperformed blue dye for SLN detection in skin cancer (15) and in breast cancer (16).

Until now, several studies demonstrated the technical feasibility and safety of ICG in SLN mapping in vulvar cancers, though most of them are case series characterized with methodological variations and lack of standardization. The aim of this study was to evaluate the SLN detection rate of ICG in vulvar cancer compared to the conventional technique using ^99m^Tc-nanocolloid and blue dye in a large cohort of patients and to analyze its applicability in different risk groups.

## Materials and Methods

We retrospectively investigated patients with histologically proven vulvar cancer who were operated at the certified cancer center of the Bern University Hospital, Switzerland between April 2013 and April 2020. The experimental protocols was approved by the Ethics Commission of the Canton of Bern, Switzerland (reference number: 261/2015) and meets the guidelines of the responsible governmental agency. All patients signed informed consent. Demographic, clinical, and intraoperative data were retrieved from an electronic database.

### Surgical Procedure

**Figure 1:**
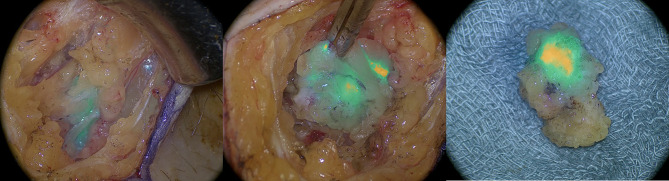
Intraoperative imaging of indocyanine green positive lymphatic channels and inguinal sentinel lymph node with near infrared imaging.

All patients underwent inguinal SLN mapping using near-infrared fluorescence imaging with ICG, applying video telescope operating microscope system technology (VITOM ICG^®^ by Karl Storz GmbH, Germany) (**Figure 1**). In every case, at least one additional tracer (^99m^Tc-nanocolloid and/or patent blue) was used. After skin incision, a gentle dissection of the fatty tissue was performed. Under near-infrared imaging, the groin was inspected for fluorescence. The groin was tested systematically for radioactivity using a handheld gamma probe and, if applicable, for blue staining by visual inspection. In accordance with international guidelines, a bilateral SLN biopsy was performed if the tumor site was located 1 cm or less from the midline. All ICG, ^99m^Tc-nanocolloid, or patent blue-positive lymph nodes were excised and sent for frozen section. If frozen section analysis revealed lymph node metastases, a complete inguinofemoral lymphadenectomy was performed. Following the SLN extirpation procedure, tumor excision was performed, consisting of a radical local excision or a vulvectomy, in function of the size and location of the tumor. Surgeries were undertaken by a team of three experienced gynecologic oncologists. For final pathology, ultrastaging of all SLN was performed.

### Sentinel Lymph Node Marking Techniques

Injection of ^99m^Tc-nanocolloid: A SLN scintigraphy was performed one day before surgery. Four aliquots of 15 MBq ^99m^Tc-labeled nanocolloids (Nano-HSA^®^, produced by Rotop Pharmaka GmbH, Dresden, Germany, particle size ≤80 nm) were injected intradermally adjacent to the tumor. After this procedure, a single-photon emission computed tomography combined with conventional computed tomography (SPECT/CT) was carried out.

**Figure 2:**
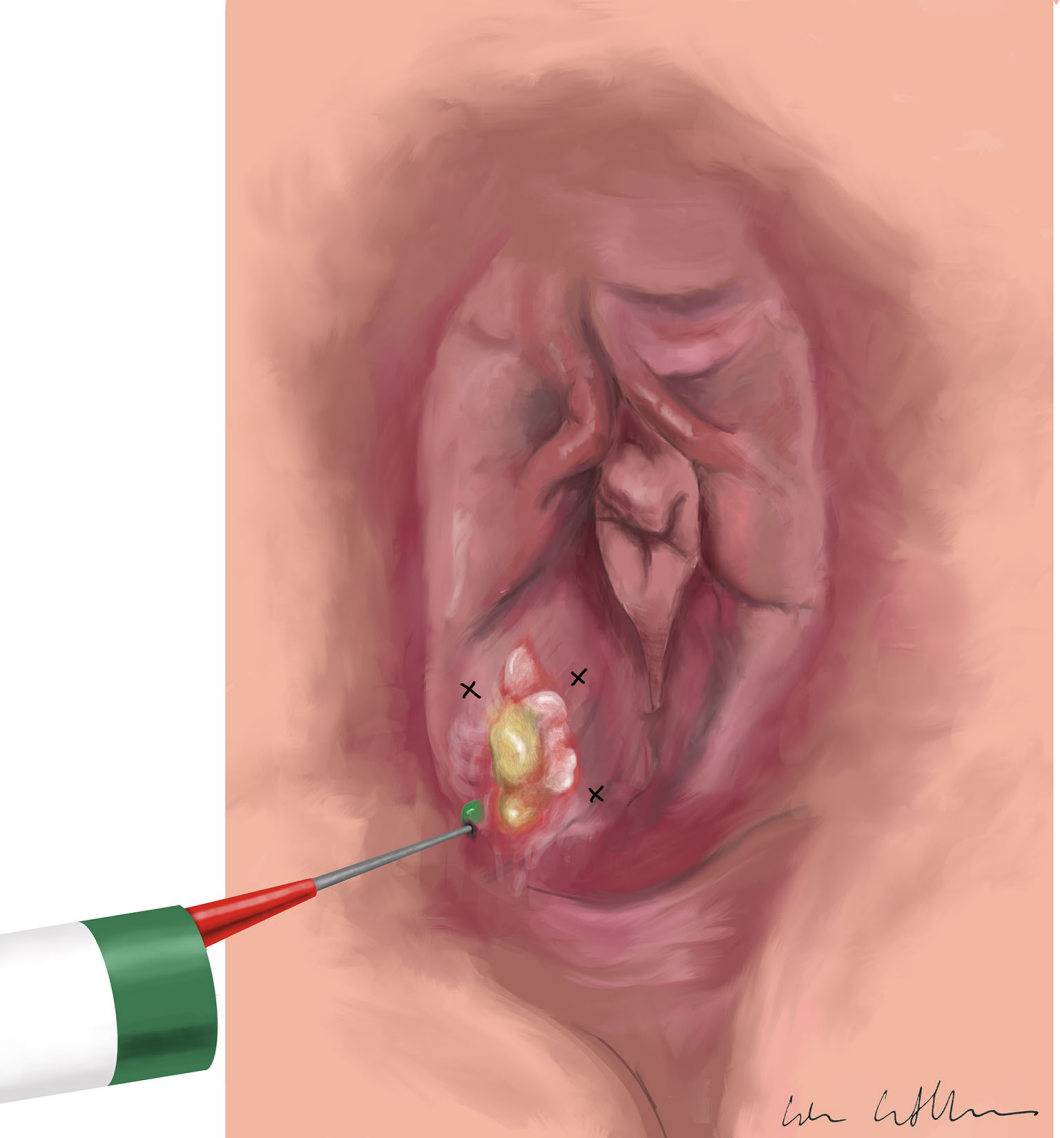
Injection of indocyanine green intradermally at four injection sites around the tumor.

Injection of ICG: One vial of 25mg ICG powder (Verdye^®^, produced by Diagnostic Green GmbH, Germany) was suspended in 10 ml of sterile water and injected intradermally directly before surgery at four injection sites around the tumor (**Figure 2**).

Injection of patent blue: A peritumoral intradermal injection of a total amount of 4ml patent blue (Patentblau V Guerbet^®^ 25mg/ml, produced by Guerbet AG, Zurich, Switzerland) was performed immediately before surgery.

### Statistical Analysis

A false negative SLN was defined as a SLN with negative tumor involvement detected with one SLN mapping technique in combination with a metastatic SLN detected with another SLN mapping technique or a metastatic non-SLN. The SLN detection rate was calculated for each SLN mapping technique, defined as the number of procedures in which at least one SLN was identified divided by the total number of procedures performed. Detection rates among the different subgroups were compared using the chi-square test. Statistical calculations were performed using the Statistical Package for Social Sciences (IBM SPSS Statistic Version 25.0).

In accordance with the journal’s guidelines, we will provide our data for the reproducibility of this study in other centers if this is requested.

## Results

Between April 2013 and April 2020, 34 patients were analyzed retrospectively for this study. Patient demographics and operative data are presented in **Table 1**. The majority of the patients had FIGO stage IB disease with a median age of 71.0 years and a median body mass index (BMI) of 27.85 kg/m^2^. The median tumor size in final pathology was 2.50 cm with a median depth of infiltration of 5.50 mm. Final histology was a squamous epithelial carcinoma in all of the cases. Adjuvant treatment was performed in eight patients. Groin recurrence rate was 2.9% with a mean follow up time of 29.9 months.

**Table 1:** Patients’ characteristics and operative data.

Median age, years (range)	70.00 (44)
Median body mass index, kg/m^2^ (range)	27.85 (23.8)
FIGO stage, n (%)	
• IA	1 (2.9)
• IB	22 (64.7)
• IIIA	5 (14.7)
• IIIB	1 (2.9)
• IIIC	4 (11.8)
• IVA	1 (2.9)
Tumor grading, n (%)	
• 1	6 (17.6)
• 2	20 (58.8)
• 3	7 (20.6)
Median operating time, min (range)	159.0 (274)
Median blood loss, ml (range)	100.0 (350)
Intraoperative complications, n (%)	0 (0)
Major postoperative complications, n (%)	3 (8.9)
Postsurgical treatment, n (%)	
• Chemoradiation	6 (17.6)
• Inguinal radiation only	2 (5.8)

n, number of patients.

Of the 34 patients, SLN mapping was performed in 64 groins with 30 patients having bilateral SLN biopsy and four patients having only unilateral. The mean amount of ICG injected was 8.4 ml (range 5 to 10 ml). No intra- or postoperative complications occurred due to the administration of ICG. In 51 groins (79.7%), a SLN biopsy alone was performed while in 13 groins (20.3%) an additional complete inguinofemoral lymphadenectomy was performed. The mean number of SLNs per groin removed was 1.88. In addition to using ICG for SLN mapping, in 53 groins we used ^99m^Tc-nanocolloid, in four groins patent blue, and in five groins both methods. In total, 120 SLNs were identified and removed, of which 103 (85.8%) were positive for ICG. In 10 groins (15.6%), we found lymph node metastases; in eight of these a SLN was detected. In seven groins no further positive lymph nodes were identified at final pathology in addition to the SLN, while in one groin two positive non-SLNs were detected in the final pathology of the complete lymph node dissection. No additional SLN was found to have metastatic disease using ultrastaging in the final pathology. In one patient (both groins affected), SLN mapping was unsuccessful using each of the three techniques. This patient therefore underwent bilateral complete inguinofemoral lymph node dissection. No false negative sentinel lymph nodes were recorded with ICG in the 13 patients who underwent complete lymphadenectomy.

The SLN detection rate of ICG (87.5%) was comparable to ^99m^Tc-nanocolloid (89.7%, p=.143) and significantly higher than patent blue (77.8%, p=0.003) (**Table 2**). The best detection rates were achieved using a combination of ICG and ^99m^Tc-nanocolloid (96.9%).

**Table 2:** Sentinel lymph node detection rates among different sentinel lymph node mapping techniques.

	SLN detection rate (%)
ICG (n=64)	87.5
^99m^Tc (n=58)	89.7
Patent blue (n=9)	77.8
ICG + ^99m^Tc (n=58)	96.9

SLN, sentinel lymph node; ICG, indocyanine green; ^99m^Tc, Technetium-99m; n, number of groins.

### Risk Group Analysis

In 19 groins the tumors showed lymph vascular space invasion and in 10 groins showed positive lymph nodes. In these cases, the SLN detection rate of ICG was significantly higher than that of ^99m^Tc-nanocolloid (p values of .004 and .035 respectively) (**Table 3**). Furthermore, we observed a higher detection rate of ICG compared to ^99m^Tc-nanocolloid in obese patients (BMI > 30 kg/m^2^), although statistically not significant (p=.707).

**Table 3:** Sentinel lymph node detection rates among different risk groups.

	SLN detection rate ICG (%)	SLN detection rate ^99m^Tc (%)	p-value
Positive lymph nodes	80.0	62.5	0.035
Lymphatic space invasion	89.5	78.9	0.004
Obesity	94.7	88.2	0.707

SLN, sentinel lymph node; ICG, indocyanine green; ^99m^Tc, Technetium-99m.

## Discussion

Accurate SLN mapping is a crucial part of vulvar cancer staging, and enables avoiding unnecessary inguinofemoral lymphadenectomies. The current standard SLN procedure consists of a combination of a radioactive tracer and a blue dye. The SLN detection rates reported in the literature are 63-82%, 88-96%, and 91-98% for blue dye, ^99m^Tc-nanocolloid, and the combination of both, respectively (17–19). Although these techniques show reasonable results in terms of detection and false negative rates, they have some shortcomings, including painful injections, complex logistics, and allergic reactions. SLN mapping with near-infrared fluorescence imaging has recently gained popularity in gynecological cancers (11, 13). Advantages include easier application, absence of radioactivity, and fewer side effects.

In our study, SLN mapping with ICG and near-infrared fluorescence imaging in vulvar cancer was feasible and safe. SLN detection with near-infrared fluorescence imaging performed equally well as ^99m^Tc-nanocolloid (87.5% vs 89.7%, p=0.143) and significantly better than patent blue alone (87.5% vs 77.8%, p= 0.003); the best results were achieved using a combination of ICG and ^99m^Tc-nanocolloid (96.9%). In patients with lymph node metastases or lymph vascular space invasion, ICG alone outperformed ^99m^Tc-nanocolloid, with a significantly higher detection rate. For the conventional SLN mapping techniques, a compromised detection rate in lymph node positive patients is described in the literature, as a result of the complete replacement of true SNL by tumor cells and a redirection of the lymphatic vessels to other nodes (17, 20). However, particularly in these patients, a reliable SLN mapping is of utmost importance. 2019 Frumovitz et al. described a superior detection rate of ICG compared to blue dye in case of metastatic sentinel lymph nodes in endometrial and cervical cancer patients (21). Furthermore, several studies describe a restricted application for ICG in obese patients due to its limited tissue penetration, with an increased BMI identified as a potential risk factor for failure in SLN mapping (22–25). Results obtained from our cohort do not support this assumption, as the detection rates of ICG and ^99m^Tc-nanocolloid did not differ significantly in obese patients, even with a slight tendency towards a higher detection rate with ICG in obese patients (94.7 vs 88.2%, p=0.707). Over all groin recurrence rate was 2.9%, which is consistent with the literature (6). The only patient with recurrence was successfully mapped with ICG and ^99m^Tc-nanocolloid revealing two negative SLNs.

Up to now, several studies reported reasonable results in ICG SLN mapping for vulvar cancer patients (**Table 4**). The largest cohort was described by Broach et al. with ICG SLN mapping in 85 patients with different histological subtypes of vulvar cancer, including melanomas and less frequent tumors (26). The further studies are mainly case series of fewer than 20 patients (22, 24, 27–30), with methodological variations. For instance, ICG was administered in different formats: either absorbed in human serum albumin (23, 27) or as the hybrid tracer ICG-^99m^Tc-nanocolloid (28, 30). Different near-infrared fluorescence imaging devices were applied, some of which were custom made (22, 29) and others commercially available [VITOM^®^ II ICG exoscope (24, 31), the Mini-FLARE™ imaging system (23, 27, 28), Photodynamic Eye (30)]. In one exploratory study, the imaging device was changed during the course of the study from SPY^®^ to PinPoint^®^ (32). In addition, few case reports have been published on feasibility and safety of robot-assisted SLN mapping with ICG in vulvar cancer patients (33, 34). This minimally invasive approach might be a valid option to further reduce short- and long-term morbidity in these patients. However, follow-up data on a larger cohort of patients are needed. Several studies focused on the sensitivity of ICG compared to ^99m^Tc-nanocolloid (23, 27, 30, 31). After injection, ICG travels *via* lymphatic vessels to the SLN as well as to echelon and second-echelon lymph nodes, potentially leading to the removal of additional, non-SLNs. Therefore, the number of lymph nodes removed is less important: the crucial point is removing the right lymph nodes. In cervical and endometrial cancer, a retrospective analysis demonstrated that a higher SLN count did not seem to increase the accuracy of SLN mapping (35). In our opinion, the SLN detection rate is the more reliable variable to investigate. Another important test characteristic is the false negative rate. As the majority of our patients did not undergo complete lymphadenectomy, we are not able to establish a false negative rate with our data.

**Table 4:** Studies using indocyanine green for sentinel lymph node mapping in vulvar cancer patients.

Author, year of publication	Type of study	No of patients	Histologic subtype	SLN marking tracers	Imaging system	ICG SLN detection rate
Broach et al. (26)	Retrospective cohort study	85	SCC Melanoma Others	ICG ^99m^Tc Blue dye	NR	96.3%
Buda et al. (24)	Retrospective cohort study	6	NR	ICG ^99m^Tc	VITOM-ICG^®^	100%
Hutteman et al. (27)	NR	9	SCC	ICG-HSA ^99m^Tc Blue dye	Mini-Flare™	NR
Verbeek et al. (28)	Prospective trial	12	NR	ICG-^99m^Tc Blue dye	Mini-Flare™	100%
Crane et al. (22)	Feasibility pilot study	10	SCC	ICG ^99m^Tc Blue dye	Custom-made	NR
Laios et al. (29)	Prospective pilot study	11	NR	ICG Blue dye	Custom-made	91%
Mathéron et al. (30)	NR	15	SCC Melanoma	ICG-^99m^Tc Blue dye	Photodynamic Eye	NR
Schaafsma et al. (23)	Double-blind randomized trial	24	SCC	ICG-HSA ^99m^Tc Blue dye ICG	Mini-Flare™	63%
Soergel et al. (31)	Prospective trial	27	NR	ICG ^99m^Tc Blue dye	VITOM-ICG^®^	NR
Prader et al. (32)	Exploratory study	33	SCC	ICG ^99m^Tc	SPY PinPoint	87.5%

No, Number; SLN, sentinel lymph node; ICG, indocyanine green; SCC, squamous cell carcinoma; ^99m^Tc, Technetium-99m; ICG-HSA, ICG adsorbed to human serum albumin; ICG-^99m^Tc, combined tracer of indocyanine green and Technetium-99m.

To our knowledge, this study contains one of the largest cohort of ICG SLN mapping in squamous cell vulvar cancer patients to date. Beside its relatively large sample size, its major strengths include the risk group analysis of patients. This research adds to a growing body of literature supporting the use of ICG in SLN mapping in vulvar cancer patients. One of its most interesting aspects is the improvement of the SLN detection rate using a combination of ^99m^Tc-nanocolloid with ICG. Based on our findings, a combination of ICG and ^99m^Tc-nanocolloid offers a reasonable alternative to the conventional SLN mapping techniques in vulvar cancer. However, the major limitation of our study is the inability to determine if ICG alone improves the SLN detection rate; specifically the value of the additional information of the SPECT/CT performed preoperatively cannot be defined in this setting.

## Data Availability Statement

The raw data supporting the conclusions of this article will be made available by the authors, without undue reservation.

## Ethics Statement

The studies involving human participants were reviewed and approved by Ethics Commission of the Canton of Bern, Switzerland. The patients/participants provided their written informed consent to participate in this study.

## Author Contributions

SI, AP and MM contributed to conception and design of the study. SM and LK organized the database. FS and SI performed the statistical analysis. FS wrote the manuscript. All authors contributed to the article and approved the submitted version.

## Conflict of Interest

The authors declare that the research was conducted in the absence of any commercial or financial relationships that could be construed as a potential conflict of interest.
